# Metallosis‐Induced Conversion Shoulder Arthroplasty: A Unique Experience and Literature Review

**DOI:** 10.1111/os.13832

**Published:** 2023-08-01

**Authors:** Jong‐Hun Ji, Sang‐Eun Park, Darshil Parikh, Woojin Lee, Jinyoung Jeong, Hyun Woo Park, Seungbae Oh

**Affiliations:** ^1^ Department of Orthopaedic Surgery, Daejeon St. Mary's Hospital College of Medicine, The Catholic University of Korea Seoul Republic of Korea; ^2^ Department of Orthopaedic Surgery, St. Vincent's Hospital College of Medicine, The Catholic University of Korea Seoul Republic of Korea

**Keywords:** Reverse, Total Shoulder Arthroplastymetallosis, Prosthesis Loosening, Rotator Cuff Tears, Total Shoulder Arthroplasty

## Abstract

**Background:**

Total shoulder arthroplasty (TSA) can fail for several reasons, such as component loosening, periprosthetic fracture, instability, infection, soft tissue failure, or joint overstuffing. Severe metallosis without loose glenoid components after TSA may result in the need for revision to reverse TSA.

**Case presentation:**

Four years before the current presentation, an 86‐year‐old woman suffered from right shoulder pain and swelling. The initial diagnosis was osteoarthritis of the shoulder joint, for which she underwent TSA. Four years later, she complained of shoulder joint pain, swelling, and limited range of motion. On sonography, subscapularis and supraspinatus tendon tears were identified. Plain radiographs and computed tomography (CT) scans showed metallosis around the shoulder joint. Due to the rocking horse mechanism, wear of the upper portion of the glenoid component and bearing caused a foreign‐body reaction and severe metallosis around the joint. Due to a massive rotator cuff tear combined with glenoid component wear, the patient eventually underwent reverse TSA (RTSA) and was satisfied with the final results.

**Conclusions:**

Severe metallosis due to glenoid component wear combined with a massive rotator cuff tear in TSA may cause the need for revision to RTSA.

## Graphical abstract

Metallosis is a possible complication after total shoulder arthroplasty without loose glenoid components. Severe metallosis due to glenoid component wear combined with a massive rotator cuff tear may result in the need for conversion to reverse total shoulder arthroplasty.

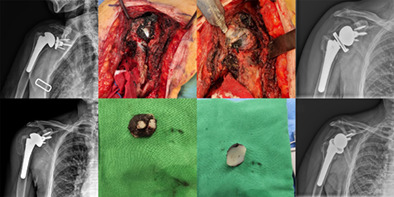

## Introduction

In Asian people with smaller humeral head size compared to Caucasian people, shoulder treatment with a larger humeral head and glenoid baseplate with polyethylene liner can cause overstuffing of the glenohumeral joint that can result in tears of both supraspinatus and subscapularis tendons.^1^ Due to such a rotator cuff tear, the rocking horse phenomenon of the humeral head can damage the superior part of the baseplate in conventional total shoulder arthroplasty (TSA) and erode the baseplate.^2^ This wear can cause severe metallosis in the shoulder joint.^3^, ^4^ Metallosis is caused by the accumulation of metallic particles around the implant components and induces an inflammatory reaction and the formation of foreign‐body giant cells and granulation tissue.^5^ This hypersensitivity reaction can lead to aseptic loosening.^6^ Overstuffing of the glenohumeral joint should be avoided in Asian populations with a smaller humeral head to prevent metallosis. The aim of the study is to report the revision of an anatomical TSA to reverse TSA due to severe metallosis following glenoid component wear combined with a massive rotator cuff tear.

## Case Report

**Fig. 1:**
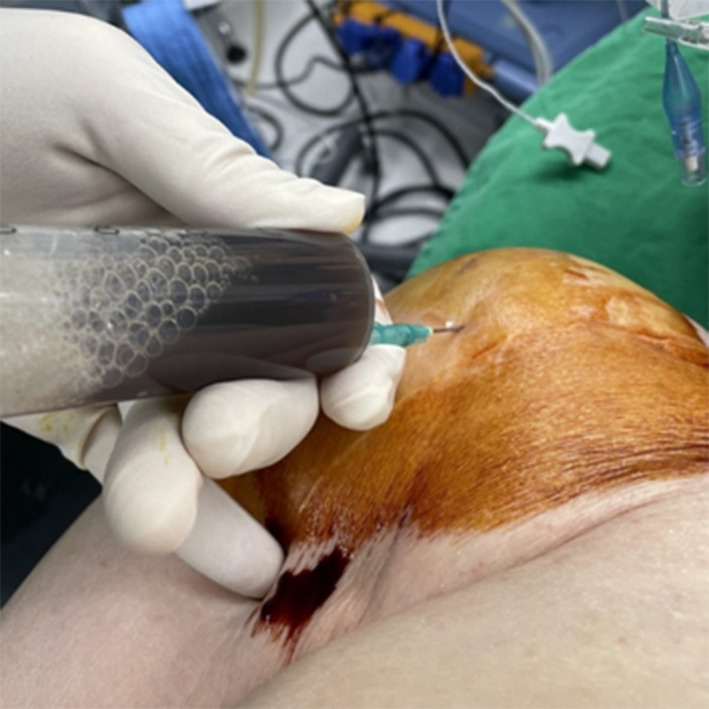
Eighty milliliters of dark‐colored joint fluid was aspirated from the shoulder joint.

An 86‐year‐old woman visited our outpatient department with sudden‐onset pain in her dominant right shoulder that had developed after daily activity without specific trauma history. Her pain was severely aggravated with shoulder movement. The pain was dull in nature, continued for 3 weeks, and was aggravated 2 days prior to hospital presentation. In her medical history, she had undergone TSA for right shoulder osteoarthritis at our hospital 4 years prior. She had not received acupuncture or intra‐articular injections to the shoulder. Upon presentation in our outpatient clinic, there was severe swelling of the shoulder joint, with mild tenderness but no localized increase in temperature. We aspirated 80 mL of dark‐colored fluid from the shoulder joint (Figure 1). The patient had a medical history of hypertension and was taking rivaroxaban for atrial fibrillation.

**Fig. 2:**
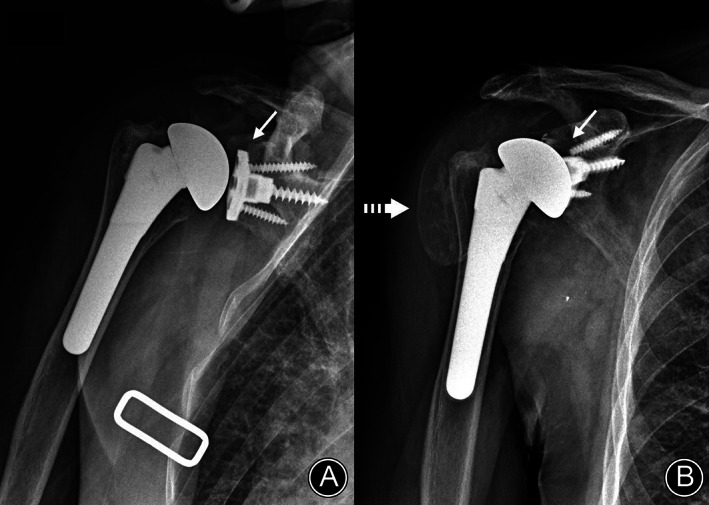
X‐ray of metallosis in the shoulder joint: (A) postoperative X‐ray showed normal component positioning without any loosening (arrow); and (B) on X‐ray 4.6 years after surgery, severe metallosis (metallic staining in the shoulder joint) was found around the shoulder joint (dotted arrow) and in the upper one‐third of the glenoid basement component that was worn out (arrow) by the anterosuperior escape of the humeral head.

**Fig. 3:**
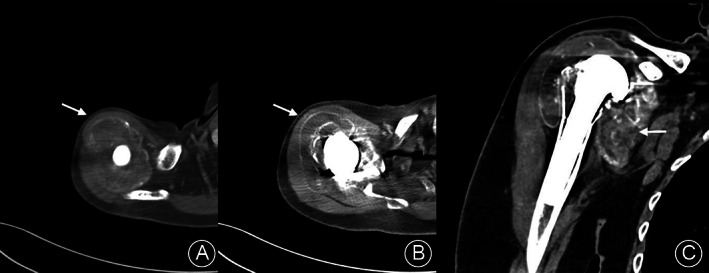
Contrast‐enhanced computed tomography scans showed rim‐enhanced and non‐enhanced fluid collection around the sub‐acromial bursa (A, arrow), subdeltoid (B, arrow), and glenohumeral joint (C, arrow).

Simple radiologic findings of the shoulder joint showed no bony abnormality. However, plain radiographs showed severe joint swelling and metallosis around the shoulder joint (metallic staining in the shoulder joint). X‐rays showed the need for reverse TSA (RTSA), with the upper one‐third of the glenoid baseplate worn and a broken screw (Figure 2). Contrast‐enhanced computed tomography (CT) showed rim‐enhanced and non‐enhanced fluid collection around the subdeltoid and subacromial bursa and around the glenohumeral joint (Figure 3), which was diagnosed as an infected fluid sac. Additionally, a low‐density lesion was observed in the fluid sac at the glenohumeral joint level. Preoperative laboratory findings of the erythrocyte sedimentation rate and C‐reactive protein were 12 mm/h and 0.28 mg/dL, respectively, and the white blood cell count of the joint fluid was 980 cells with a greenish color.

**Fig. 4:**
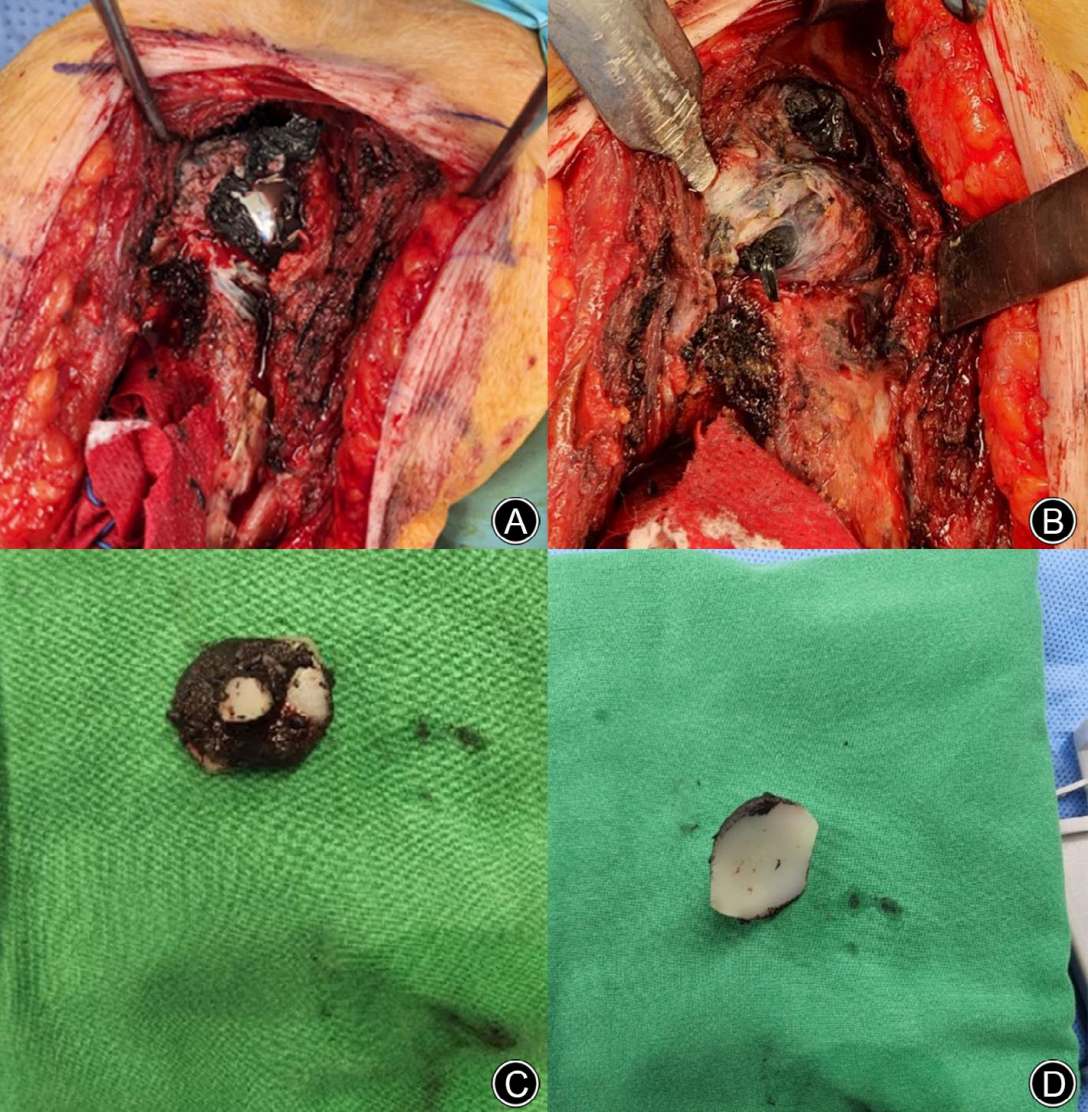
Intraoperative findings: (A) thin and elongated subscapularis tendon was found; and (B) torn subscapularis and supraspinatus tendons are shown, with metallosis debris shown in the glenohumeral joint and proximal humerus; (C) wear of the upper portion of the glenoid component is shown clearly in the glenohumeral joint; and (D) the glenoid bearing is worn out and already displaced from the baseplate.

**Fig. 5:**
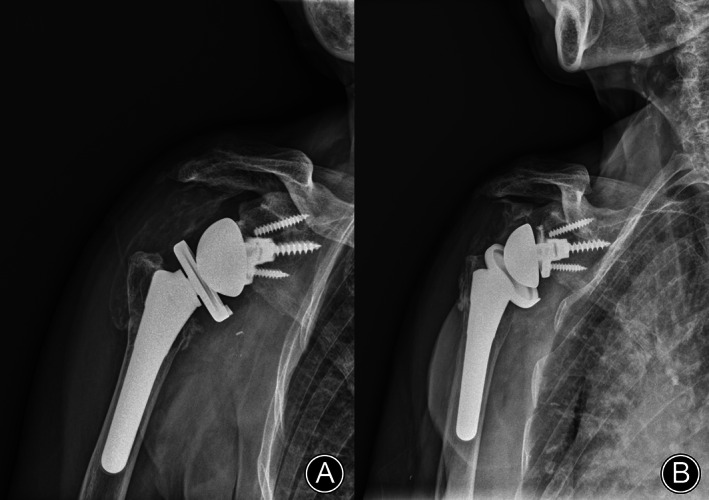
Postoperative X‐ray: (A) conversion of total shoulder arthroplasty to reverse shoulder arthroplasty with convertible arthroplasty system; and (B) the follow‐up X‐ray performed 1 year after surgery showed normal component positioning without any loosening.

Intraoperative findings were as follows. Following a previous incision, the deltopectoral approach was performed. A needle‐tip electrocautery was used to dissect subcutaneous or deep soft scar dissection to prevent hemorrhage. The cephalic vein was not identified during the revision surgery. After dividing the deltoid and pectoralis major muscle, we tried to find the biceps tendon that was attached to the bicipital groove during the previous surgery. After searching the bicipital tendon and glenohumeral joint, scar tissue and adhesion were carefully removed. A torn subscapularis tendon and metallosis debris were observed in the glenohumeral joint and proximal humerus. The glenoid bearing was worn out and had displaced from the baseplate, and wear of the upper portion of the glenoid component was evident in the glenohumeral joint (Figure 4). Complete debridement and thorough irrigation of the metallosis were performed. Even though the upper one‐third of the glenoid baseplate was worn out, a glenosphere was inserted without instability (Figure 5). Postoperatively, pendulum exercise was started immediately after recovery, and an ultra‐arm sling was used for 4 weeks. The patient's most recent follow‐up was at 1 year. She had a pain visual analogue scale (VAS) score of 2, an American Shoulder and Elbow Surgeons (ASES) score of 85, a University of California, Los Angeles (UCLA) score of 31, a Simple Shoulder Test (SST) score of 10, and a Korean Shoulder Score (KSS) of 88. The range of motion was forward flexion 150°, abduction 140°, and external rotation 35°. The internal rotation level was L1 behind the back. The patient was eventually satisfied with the results.

## Discussion

### Metallosis and Revision to Reverse Total Shoulder Arthroplasty

In this case, severe metallosis following TSA 4 years prior developed without loose glenoid components. This patient presented with severe metallosis and eventually underwent reverse TSA due to glenoid component wear combined with a massive rotator cuff tear. Although there was severe metallosis due to the glenoid component wear, TSA was converted to reverse shoulder arthroplasty with a convertible arthroplasty system.

Metallosis is a condition resulting from metal‐on‐metal contact with periprosthetic tissues that leads to the release of metal ions, which then bind to native proteins, causing a hypersensitivity reaction.^5^, ^7^ This reaction leads to an inflammatory response resulting in the recruitment of macrophages and formation of granulation tissue.^8^, ^9^ Typically, metallosis occurs after total hip arthroplasty and total knee arthroplasty.^10^, ^11^ However, it has also been reported in non‐weight bearing joints like the elbow and shoulder.^5^, ^12^ Diagnosis of metallosis cannot be made radiographically, as imaging has not been able to reliably demonstrate its presence.^13^ However, X‐ray and computed tomography (CT) scans showed typical metallosis of the shoulder joint (with metallic staining in the shoulder joint). TSA recreates the normal anatomy of the shoulder ball and socket joint, placing the ball on the humeral component and creating a socket in the glenoid component. During TSA revision surgery, the conversion of failed shoulder arthroplasty to reverse TSA (RTSA) has become common.^14^ Many commercially available TSA systems now offer a platform humeral stem or glenoid sphere that is used for both anatomic shoulder arthroplasty and RTSA.^4^, ^15^

### Causative Factors of Revision

Total shoulder arthroplasty can fail for several reasons, such as component loosening, periprosthetic fracture, instability, infection, soft tissue failure, or joint overstuffing.^5^, ^13^, ^16^, ^17^ Metallosis‐induced conversion arthroplasty has been reported very rarely^3^, ^4^ (Table 1). During surgery, severe greenish metallic staining was found in the joint, confirming the diagnosis of metallosis, and diffuse wear was observed in the polyethylene insert. This was probably due to instability due to early hypermobility, reduced tension on the deltoid muscle, inadequate liner size, downward tilt of the glenoid, and failure to lateralize the component sufficiently.^5^

**TABLE 1:** Literature review of the cases with metallosis‐induced conversion arthroplasty

No.	Author	Sex/Age Location	Diagnosis	Initial arthroplasty	Conversion arthroplasty
1	Khan *et al*.^3^	M/55 Rt. shoulder	Destructive rheumatoid arthritis	Total shoulder replacement arthroplasty (Nottingham)	Total shoulder replacement arthroplasty (TSA)
2	Matsoukis *et al*.^4^	M/71 Lt. shoulder	Unknown	Total shoulder replacement arthroplasty (TSA)	Reverse total shoulder replacement arthroplasty (RTSA)

Loosening of the implant was suspected, and the patient required a repeat operation. In the operation field, no component loosening was identified, especially in the glenoid component. Although the upper portion of the glenoid baseplate was worn, there was no baseplate loosening. Implant metallosis was identified and treated with conversion to RTSA.

An important complication after anatomic TSA is iatrogenic rotator cuff injury or attritional rotator cuff tears.^17^ Such injuries can occur if humeral neck osteotomy is inferior to the level of rotator cuff insertion. Additionally, overstuffing of the glenohumeral joint could lead to attritional supraspinatus and subscapularis tears, which are common reasons for conversion to RTSA. The present case was treated using the convertible anatomic TSA system, which offers flexibility to convert from a hemiarthroplasty or total arthroplasty to a reverse shoulder procedure and allows later revisions if necessary. Using this system, humeral stem retention was associated with a significantly shorter operative time.

### Suggestions Based on Experience

The system used has 17 head sizes ranging from 38 to 58 mm in diameter and 18 to 27 mm in height. In Korea, a 38‐mm head diameter only provides 19 or 21 mm of height. A component composed of a head size that was 38 mm in diameter and 19 mm in height was used for the present case. In Asian people with smaller humeral head sizes than Caucasian people, this head height is excessive and can result in subscapularis tears and supraspinatus tendon tears with even minor trauma. In our patient, the larger humeral head and glenoid baseplate with polyethylene liner caused overstuffing of the glenohumeral joint, resulting in tears in both supraspinatus and subscapularis tendons. Initial subscapularis tears can extend to the supraspinatus tendon *via* the zipper phenomenon. In our patient, owing to both supraspinatus and subscapularis tears, the humeral head migrated upward, and wear of the upper portion of the glenoid baseplate occurred due to the rocking horse mechanism. After the subscapularis tear, the humeral head of the TSA moved to the anterosuperior area. After bearing wear, the upper portion of the glenoid base was also eroded. X‐ray and CT images showed subscapularis and rotator cuff tears and wear to the upper portion of the glenoid component and bearing, which led to metallosis due to the rocking horse mechanism and possibly overstuffing or the large size of the humerus head. The patient underwent RTSA. Overstuffing of the glenohumeral joint should be avoided in Asian populations with smaller humeral heads. Care must be taken when performing TSA to ensure the humeral component is not oversized. When a subscapularis tenotomy is performed, brace maintenance is recommended for a certain period to allow for subscapularis tendon healing.

### Conclusion

Metallosis is a possible complication after TSA without loose glenoid components. Severe metallosis due to glenoid component wear combined with a massive rotator cuff tear may cause the need for revision to reverse TSA.

## Author Contributions

The authors meet the authorship criteria according to the latest guidelines of the International Committee of Medical Journal Editors and are in agreement with the manuscript.

## Conflict of Interest Statement

The authors declare no potential conflicts of interest with respect to the research, authorship, and/or publication of this report.
